# The investigation of ^125^I seed implantation as a salvage modality for unresectable pancreatic carcinoma

**DOI:** 10.1186/1756-9966-32-106

**Published:** 2013-12-27

**Authors:** Hao Wang, Junjie Wang, Yuliang Jiang, Jinna Li, Suqing Tian, Weiqiang Ran, Dianrong Xiu, Yang Gao

**Affiliations:** 1 Department of Radiation Oncology, Peking University 3rd Hospital, Huan-yuan North Road 49th, Beijing 100083, P. R. China; 2 Department of Ultrasound, Peking University 3rd Hospital, Beijing 100083, P. R. China; 3 Department of Surgery, Peking University 3rd Hospital, Beijing 100083, P. R. China

**Keywords:** ^125^I seed, Intraoperative implantation, Ultrasound-guided, Unresectable, Pancreatic carcinoma

## Abstract

**Background:**

To assess the efficacy of intraoperative ultrasound-guided implantation of ^125^I seeds for the treatment of unresectable pancreatic carcinoma, and analyze the associated prognostic factors.

**Methods:**

Twenty-eight patients with pancreatic carcinoma who underwent laparotomy and were considered to have unresectable tumors were included in this study. Nine patients were pathologically diagnosed with Stage II disease, and nineteen patients with Stage III disease. Twenty-eight patients received intraoperative ultrasound-guided ^125^I seed implantation and received a D_90_ (at least 90% of the tumor volume received the reference dose) ranging from 60 to 163 Gy, with a median of 120 Gy. Seven patients received an additional 35–50 Gy external beam radiotherapy after seed implantation, and ten patients received two to ten cycles of chemotherapy. Overall survival of the patients was calculated and prognostic factors were evaluated.

**Results:**

Of the patients, 94.1% (16/17) achieved good to medium pain relief. The tumor response rate was 78.6% (22/28), and local control was achieved in 85.7% (24/28) of patients. The 1-, 2- and 3-year survival rates were 30%, 11% and 4%, and the median survival was 10.1 months (95% CI: 9.0-10.9). Analysis using the Cox proportional hazards model suggested that patients younger than 60 years and patients who received a D_90_ higher than 110 Gy may survive for a longer period.

**Conclusions:**

I seed implantation provides a safe and effective method to relieve pain, control local tumor growth and, to some extent, prolong the survival of patients with stage II and III pancreatic disease, without additional complications. Age and accumulated dose may be factors predictive of a favorable outcome for patients with unresectable pancreatic carcinoma treated with ^125^I seeds. These findings need to be validated by conducting further studies with larger cohorts.

## Background

Pancreatic carcinoma is the tenth most common malignant tumor, but is the fourth most common cause of cancer-related deaths worldwide [1]. Less than 20% of pancreatic carcinoma patients are suitable for surgical resection, the majority of cases of pancreatic carcinoma are diagnosed at the locally advanced or metastatic stage. The median survival of locally advanced or metastatic disease is approximately 6 months with palliative treatment. But even the tumors are resected, long term survival still remains poor [2,3].

Pancreatic carcinoma survival rates have shown little improvement over the past 30 years. Despite the introduction of new therapeutic techniques combined with aggressive modalities, such as external beam radiotherapy (EBRT), intraoperative radiotherapy (IORT) and chemotherapy, the prognosis for patients with pancreatic carcinoma remains unsatisfactory, with a 5-year survival rate less than 6% [1]. At present, National Comprehensive Cancer Network guidelines recommend treatments including gemcitabine- and capecitabine-based chemotherapy or concurrent chemoradiation for patients with good performance status, resulting in a median survival of only 9.2-11.0 months [4]. Once, IORT was expected to improve the long-term survival of pancreatic cancer patients, while clinical results were not satisfactory [5,6].

Currently, there is no consensus regarding the best therapeutic modality for unresectable pancreatic carcinoma. It is necessary to investigate novel techniques that may improve patient outcome. Wang et al. were the first group to investigate the use of intraoperative ultrasound-guided ^125^I seed implantation as a new technique for managing unresectable pancreatic carcinoma, and demonstrated that the technique was feasible and safe [7]. In this study, we confirmed the efficacy of ^125^I seed implantation, and analyzed the possible factors associated with favorable clinical outcomes.

## Methods

### Characteristics of patients

Between October 2003 and August 2012, twenty eight patients with a Karnofsky performance status (KPS) score of 70 or above were identified. Of these twenty eight patients, 39% (10/28) had jaundice, 60% (17/28) suffered pain, 11% (3/28) had intestinal obstruction and 93% (26/28) experienced weight loss. These patients were diagnosed with unresectable pancreatic carcinoma by surgeons carrying out a laparotomy, and received ^125^I seed implantation guided by intraoperative ultrasound. The criteria of unresectable disease included vascular invasion, or vascular invasion combined with metastasis to the local regional lymph nodes. Of the twenty eight pancreatic carcinoma patients, nine were diagnosed with stage II disease, and nineteen patients had stage III disease. Summaries of the patients’ characteristics are listed in Table 1, Additional file 1: Table S1 and Additional file 2; Table S2. Five of the patients with jaundice received a biliary stent one month before ^125^I seed implantation. All patients were evaluated for the extent of disease progression by physical examination, complete blood panel, chest X-ray, abdominal CT scans and ultrasound prior to seed implantation. This study was approved by the institutional review board and informed consent was obtained from all patients. Institutional Review Board: Peking University Third Hospital Medical Science Research Ethics Committee.

**Table 1:** Summary of patient characteristics (n = 28)

	**No of patients**	**%**
Gender		
Male	16	57
Female	12	43
Stage II		
pT3N0M0	6	21
pT1N1M0	0	0
pT2N1M0	1	4
pT3N1M0	2	7
Stage III		
pT4N0-1 M0	19	68
Primary tumor location		
Head	15	54
Body and/or tail	11	39
Whole pancreas	2	7
Symptoms		
Jaundice	11	39
Pain	17	60
Weight loss	26	93
Pathology		
Adenocarcinoma	28	100

### Treatment planning protocol

**Figure 1:**
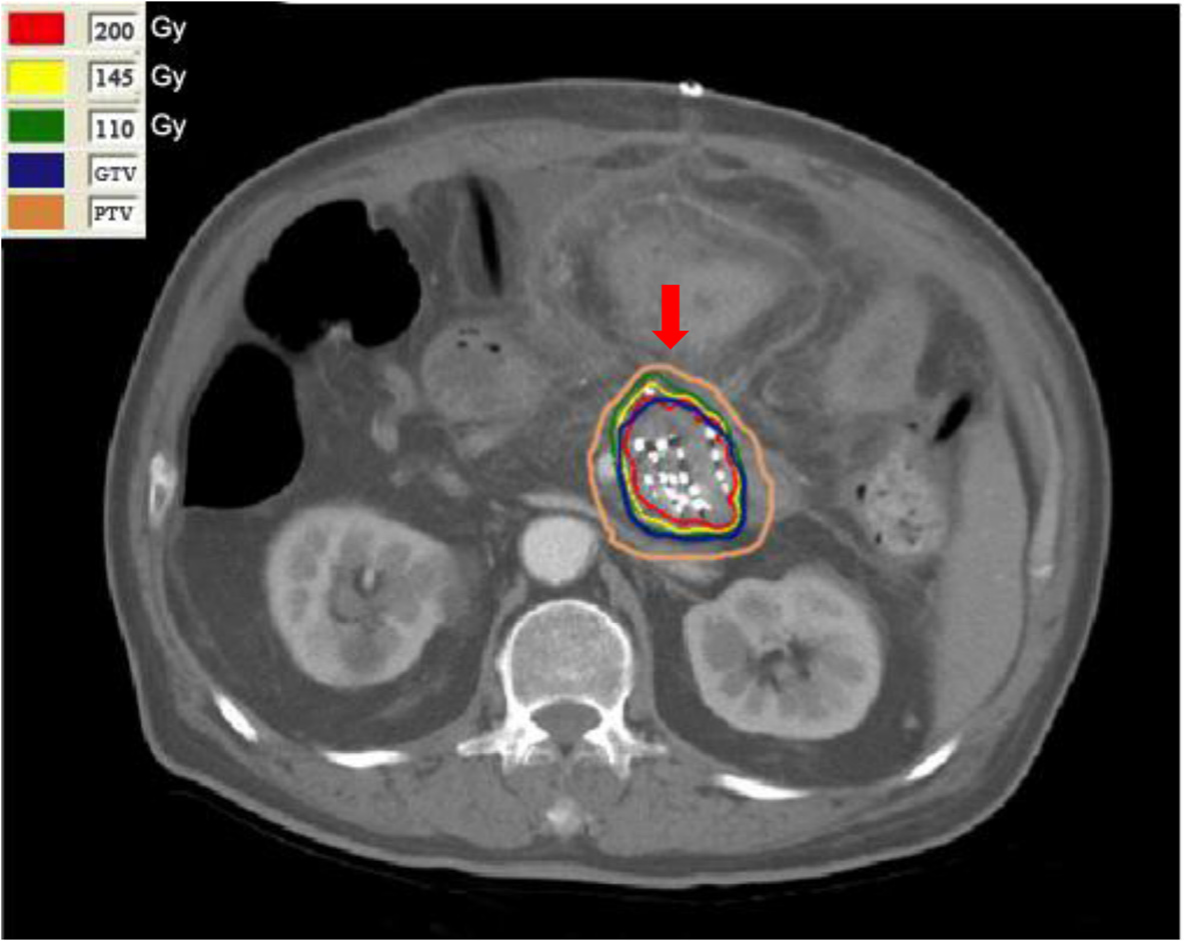
**CT image and dose distribution curves of a typical patient.** Male, 63 years old, stage III, T4N0M0. The green line is the isodose curve for 110 Gy.

Computerized tomography (CT) scans were taken 1–2 weeks before seed implantation to evaluate detailed tumor location and volume of patients. Images of pancreatic carcinomas were obtained at 5 mm intervals. The gross tumor volume (GTV) was outlined by radiation oncologists and surgeons on each image in consultation with one another. The planning target volume (PTV) included GTV plus 0.5-1.0 cm peripheral tissue. These traces were digitized and scanned to define the tumor volume, from which the D_90_ of 60–163 Gy (median 120 Gy) for ^125^I seed irradiation could be calculated. Then the system figured out the required number of ^125^I seeds to be implanted. The D_90_ was defined that at least 90% of the tumor volume received the reference dose (Figure 1). The ^125^I seeds (Beijing Atom and High Technique Industries Inc, Beijing, Model-6711) had a half-life of 59.4 days with a low energy level of 27.4 KeV and a half-value layer of 0.025 mm of lead. A computerized treatment planning system (Beijing Fei Tian Technique Industries Inc, Beijing, China) was used for dose calculations.

### Ultrasound-guided seed implantation

Following collection of an intraoperative biopsy to establish the diagnosis of pancreatic cancer, tumor volume was measured during laparotomy by intraoperative ultrasonography utilizing a megahertz linear probe. Guided by ultrasound, 18-gauge needles were implanted into the mass and spaced at intervals of 1.0 cm in a parallel array, extending at least 0.5-1.0 cm beyond the margins of the pancreatic lesions. During the placement of the needles, care was taken to avoid the needles penetrating the pancreatic duct, small blood vessels, and the adjacent transverse colon by ensuring placement at least 1 cm from these tissues. ^125^I seeds were implanted using a Mick applicator following insertion of the needles, and the spacing for seeds in the same needle is 1 cm [7]. The number of ^125^I seeds implanted ranged from ten to seventy five; the median number was thirty five. The specific activity of ^125^I seeds ranged from 0.40 to 0.60 mCi per seed, and the total isotope radioactivity implanted ranged from 4 to 37.5 mCi. An omental fat pad was placed over the implanted volume to protect the gastric and transverse colon mucosa from excessive irradiation.

### Adjuvant therapy after initial treatment

EBRT was generally recommended for all patients as an adjuvant therapy, but only seven patients received EBRT 4–6 weeks after ^125^I seed implantation. The total dose of EBRT ranged from 35 to 50 Gy at 1.8-2.0 Gy per fraction. Postoperative chemotherapy was recommended for all patients on an adjuvant or palliative basis, but only ten patients received chemotherapy consisting of gemcitabine or paclitaxel and completed two to six cycles. The remaining patients refused EBRT or chemotherapy following seed implantation.

### Definition of tumor response

Tumor response was assessed using WHO criteria [8]. In brief, a complete response (CR) was defined as the complete disappearance of all measurable lesions, without the appearance of any new lesion(s). A partial response (PR) was defined as a reduction in bidimensionally measurable lesions by at least fifty percent of the sum of the products of their largest perpendicular diameters, and an absence of progression in other lesions, without the appearance of any new lesion(s). Stable disease (SD) was defined as a reduction in tumor volume of less than fifty percent or an increase in the volume of one or more measurable lesions of less than twenty five percent, without the appearance of any new lesion(s). Progressive disease (PD) was defined as an increase in the tumor volume by at least twenty five percent and the appearance of any new lesion(s). The response rate was equal to the CR + PR.

### Pain evaluation and definition of treatment response

Pain is one of the most common clinical symptoms of pancreatic carcinoma. Pain intensity was evaluated and graded by the Numerical Rating Scale (NRS). NRS score 1–3 was defined as mild pain, NRS score 4–6 was defined as moderate pain and NRS score 7–10 was defined as severe pain [9]. A good response was defined as severe or moderate pain decreasing to no pain post-treatment, and a medium response was regarded as severe or moderate pain reducing to mild pain after treatment, with pain-free sleep and maintenance of a normal lifestyle. A poor response meant that there was no change in the severity of pain, compared with pre-seed implantation.

### Patient follow-up

Patients were evaluated by radiation oncologists and surgeons one month after seed implantation. Regular items included physical examination, complete blood panel, chest X-ray, abdominal CT and ultrasound. Then, a clinical consultation was provided, followed by evaluation every 2–3 months or sooner if a new clinical sign or symptom appeared. Survival was calculated from the date of diagnosis to the date of death, or last follow-up. Local recurrence was defined as tumor progression within the implanted area or surrounding regions according to CT images. Local recurrence and distant metastases were scored until patient death, and censored thereafter.

### Statistical analyses

Overall survival rates were estimated using the Kaplan-Meier method, and deaths for any reason were scored as events. The impact of different factors on the survival curves was analyzed using the log-rank test and Cox proportional hazards model, that were frequently used in single factor analysis and multiple factor analysis. SPSS version 16.0 was used for statistical analysis, with the level of significance defined as a *p* value of <0.05.

## Results

### Tumor local control and patients’ survival

**Figure 2:**
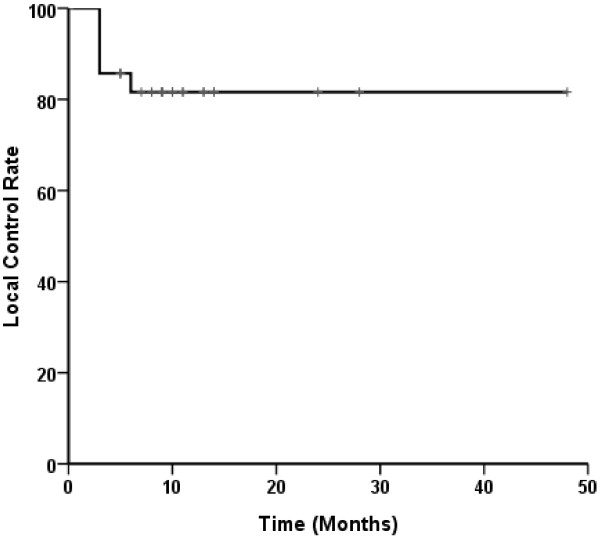
**Actuarial local control curve for twenty eight patients.** Patients with unresectable stage II/III pancreatic carcinoma were treated with ^125^I seed implantation.

**Figure 3:**
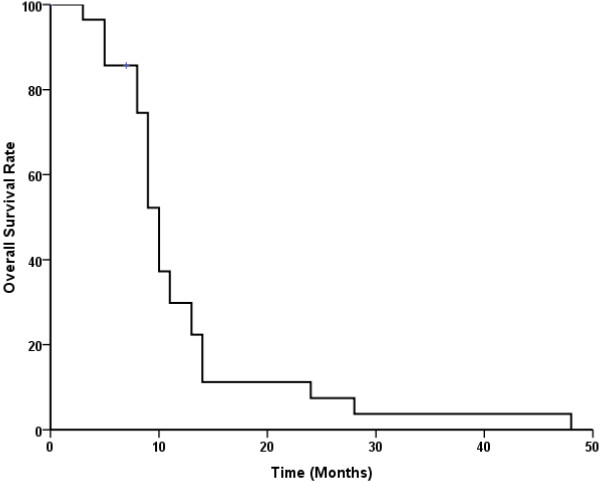
**Actuarial survival curve for twenty eight patients.** Patients with unresectable stage II/III pancreatic carcinoma were treated with ^125^I seed implantation.

In our study, the tumor response rate was 78.6%, with an overall local control rate of 85.7% (24/28) (Figure 2). The Kaplan-Meier actuarial survival curve for all twenty eight patients treated with seed implantation is shown in Figure 3. The overall 1-, 2- and 3-year survival rates were 30%, 11% and 4%, respectively. The overall median survival time was 10.1 months (95% CI, 9.0-10.9). Twenty two patients died of metastases to the liver and peritoneal surface, yet had no imaging evidence of any residual local disease. Two patients died of local progression, two patients died of local progression and metastases, one patient died of heart disease, and one patient was still alive at last follow-up.

### Pain relief

Pain is one of the most common clinical symptoms of pancreatic carcinoma. 60% (17/28) of patients were suffering pain prior to treatment, and 94.1% (16/17) of patients achieved a good or medium response after ^125^I seed implantation. Almost half of the patients (47%, 8/17) achieved good response. Three patients suffering severe pain and five patients with moderate pain were all reported no pain after treatment. An additional 47% (8/17) of patients achieved medium response. Six patients with severe pain and one patient with moderate pain were reported only mild pain following treatment. Only one patient continued to suffer moderate pain after treatment. The majority of patients experienced some relief from pain within one week following seed implantation.

### Toxicity and complications

There were few toxicity and complications, and no patients died during the perioperative period. Chylous fistula was observed in one patient (4%). Gastric ulcer was observed in one patient (4%) who underwent seed implantation and EBRT. Two patients (7%) experienced radiation enteritis and ten patients (36%) experienced transient fever. In addition, in each of two (7%) patients, three seeds were found to have migrated to the liver. However, no side effects were observed in the 12 months post-treatment.

### Prognostic factors

**Figure 4:**
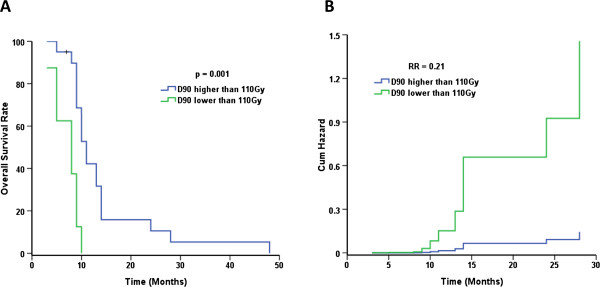
**A D**_**90 **_**higher than 110 Gy is a favorable prognostic factor.** Patients with unresectable stage II/III pancreatic carcinoma were treated with ^125^I seed implantation. The blue line is for the group whose doses were higher than 110 Gy. The green line is for the group whose doses were lower than 110 Gy. **A**. Overall survival rate curves for the two groups. **B**. Hazard function curves for the two groups.

**Figure 5:**
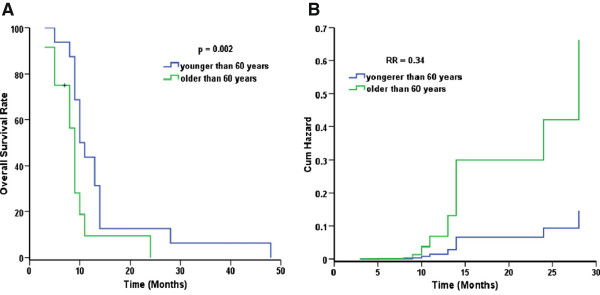
**Age younger than 60 years is a favorable prognostic factor.** Patients with unresectable stage II/III pancreatic carcinoma were treated with ^125^I seed implantation. The blue line is for the group whose ages were younger than 60 years. The green line is for the group whose doses were older than 60 years. **A**. Overall survival rate curves for the two groups. **B**. Hazard function curves for the two groups.

Multiple factors that may affect overall survival were analyzed. Log-rank single factor analysis suggested that patients who actually received a D_90_ higher than 110 Gy (calculated after seed implantation), and patients younger than 60 years may survive longer. The median survival of patients who actually received a D_90_ higher or lower than 110 Gy was 11 months (95% CI: 9.3-12.6) and 8 months (95% CI: 3.9-12.0), respectively, *p* < 0.001. The median survival of patients younger than 60 years was 10 months (95% CI: 8.0-11.9), compared with 9 months (95% CI: 8.0-9.9) for patients over 60 years old (*p* = 0.035). The outcome of patients with pancreatic carcinoma in the head of the pancreas and jaundice may be poor. The median survival time of patients with cancer in the head of the pancreas was 9 months (95% CI: 8.3-9.7) compared with 11 months (95% CI: 9.3-12.6) for patients whose tumor was situated outside of the head of the pancreas (*p* = 0.15). The median survival of patients with and without jaundice was 9 months (95% CI: 8.3-9.6) and 11 months (95% CI: 9.4-12.5), respectively (*p* = 0.09). Patients who achieved CR and received adjuvant EBRT may survive longer. However additional patients should be enrolled to verify these observations. The median survival of patients achieving CR or not was 24 months (95% CI: 7.9-40.0) and 9 months (95% CI: 8.0-9.9), respectively (*p* = 0.05). However, only three patients achieved CR, with overall survival of 14, 24 and 28 months, respectively. The median survival of patients receiving adjuvant EBRT or not was 13 months (95% CI: 8.3-17.6) and 10 months (95% CI: 9.0-10.9), respectively (*p* = 0.24). However, only seven patients received adjuvant EBRT, and six of these patients were younger than 60 years. Gender, adjuvant chemotherapy, tumor volume and CA199 level before and after the operation did not impact the clinical outcome (*p* > 0.05). The result of the Cox proportional hazards model suggested that a D_90_ higher than 110 Gy was an independent, favorable prognostic factor comparing with lower than 110 Gy (*p* = 0.001), and the relative risk ratio was 0.21 (95% CI: 0.08-0.57). The fitted curve is shown in Figure 4. Patient age younger than 60 years was another independent, favorable prognostic factor comparing with older than 60 years (*p* = 0.002), and the relative risk ratio was 0.34 (95% CI: 0.13-0.91). The fitted curve is shown in Figure 5.

## Discussion

Pancreatic cancer has an appalling prognosis, especially for patients with unresectable tumors at the time of diagnosis, which represents more than 80% of patients. Therefore the treatment of unresectable pancreatic cancer continues to be a major challenge. In approximately 30% of patients with unresectable tumors, the lesions remain locally advanced without evidence of distant metastases at autopsy [10]. Therefore, localized treatments are extremely important for tumors that are locally or regionally confined. A recent systematic review once again concluded that surgery was not an optimal choice for these patients, as morbidity and mortality rates increased after R2 resection, with pooled median survival time of only 8.2 months [11]. Radiotherapy is recommended to prolong overall survival, and improve local disease and symptom control [12].

Radiation techniques such as three-dimensional conformal radiotherapy, intensity-modulated radiotherapy (IMRT), stereotactic body radiation therapy (SBRT), intraoperative radiation therapy, and low-dose rate (LDR) or high-dose rate (HDR) radiation have all been used in the treatment of locally advanced pancreatic cancer. However, the clinical outcomes are unsatisfactory. There is evidence that common external beam radiation with or without chemotherapy can achieve a median survival time of 8.2-14.8 months, with the incidence of grade III to IV complications between 10% and 25% [13-16]. The potential benefits of SBRT alone are still controversial, due to poor patient outcome, unacceptable toxicity and questionable palliative effects. Hoyer et al. reported the results of a Phase II study using SBRT in the treatment of locally advanced pancreatic carcinoma, in which the median survival time was only 5.7 months, with 18% of patients suffering from severe mucositis or ulceration of the stomach or duodenum [17]. Recently, there have been reports suggesting that SBRT and chemotherapy might be a useful treatment option, resulting in a median survival time of 10.6-14.3 months with acceptable complications [18-20]. Additional reports suggest that IORT can be used to prevent local recurrence after resection or to control abdominal pain. However, the median survival time was 7.1-10.5 months [21,22]. Disappointingly, the combined use of IORT and EBRT also failed to significantly improve long-term survival, with a median survival time of only 7.8-11.1 months [5,6]. A report of interstitial iridium-192 HDR brachytherapy for the treatment of unresectable pancreatic carcinoma found a median survival time of 6.5 months for stage II/III in the absence of severe, acute side effects [23].

Recent years, there were some basic research indicated that ^125^I seed continuous low dose rate irradiation may be beneficial to pancreatic carcinoma. Wang et al. reported that ^125^I seeds irradiation could induce higher apoptotic rates of PANC-1 pancreatic cancer cells, which led to programmed cell death [24]. Ma et al. reported that ^125^I seed continuous low dose rate irradiation inhibited pancreatic cancer tumor growth and changed DNA methyltransferases expression patterns [25]. Gao et al. found aberrant DNA methyltransferase expression in pancreatic ductal adenocarcinoma tissue, which maybe promoted tumor development and progression [26]. Similar biological effects were found in gastric and rectal cancer cell lines [27,28]. Basic research provided evidences for ^125^I seed continuous low dose rate irradiation from beach to bedside.

The advantage of permanent interstitial radioactive seed implantation into the tumor site is the ability to deliver a high dose of irradiation to the tumor while minimizing relative exposure to the surrounding area. In some medical centers who have skillful surgeons, radiation oncologists and license of ^125^I seed implantation, intraoperative ultrasound-guided implantation of ^125^I seeds for unresectable pancreatic carcinoma is feasible and safety, especially for those centers who do not have IORT equipment. ^125^I seeds were selected as the radioactive source, due to the half-life of the isotope of 59.4 days [29]. The technique of implanting radioactive isotopes to treat pancreatic carcinoma has been used for several decades. Handley first reported the treatment of seven cases of pancreatic cancer using radium needle implantation in 1934 [30]. Hilaris, a pioneer in the development of ^125^I seed implantation for the treatment of pancreatic carcinoma, enrolled ninety eight patients, and achieved a median survival time of 7 months in 1975 [31], with one patient surviving for five years. Morrow et al. concluded that there was no difference in survival between interstitial brachytherapy and surgical resection at the same institution [32]. These results indicated that overall survival following ^125^I seed implantation was comparable with other techniques in patients with locally advanced pancreatic carcinoma [33]. Wang et al. first reported on the use of the novel technique of intraoperative ultrasound-guided ^125^I seed implantation to manage unresectable pancreatic carcinoma, and demonstrated that it was a feasible and safe technique [7]. Our study expands these findings to additional cases and confirms the efficacy and lack of complications associated with this technique. The tumor response rate was 78.6%, with an overall local control rate of 85.7% (24/28) in our cohort of patients. The overall median survival time was 10.1 months, while the overall 1-, 2- and 3-year survival rates were 30%, 11% and 4%, respectively. Ninety four percent (16/17) of patients achieved good or medium relief from pain. These data were all comparable with, or better than, the results of surgery and other radiotherapy techniques [29-33].

The limitation of permanent interstitial radioactive seed implantation in pancreatic cancer is the high rate of perioperative morbidity and mortality, since most of the earlier radioactive seed implantation techniques were performed by eye during surgery. In a previous study using eye guided implantation, the perioperative mortality rate was 16% to 25% due to acute pancreatitis, fistulization, and abscess formation [34]. Probable reasons for the high mortality may be the high incidence of penetration into the pancreatic duct, small blood vessels in the pancreas and/or adjacent organs. Wang et al. reported that under the guidance of ultrasound, the incidence of collateral damage decreased, no perioperative mortality was observed, and no grade III to IV complications were reported [7]. In this study, we confirmed that there were no operation-associated mortalities or grade III to IV complications. Only one patient suffered from chylous fistula, one patient suffered from gastritis, two patients suffered from radiation enteritis and ten patients suffered from low fever, which is lower than the incidence of complications reported in the published data of surgery and radiotherapy [34].

The data indicate that younger patients with good performance status, or treatment with gemcitabine- or capecitabine-based chemotherapy were favorable prognostic factors [35-38]. Multiple factors were analyzed using the log-rank single factor model, and the data suggested that patients who actually received a D_90_ higher than 110 Gy and patients younger than 60 years may survive longer (*p* < 0.05). The outcome of patients with pancreatic carcinoma in the head of the pancreas or who have jaundice may be poor. However, additional patients should be observed to confirm these findings. Gender, adjuvant chemotherapy, tumor volume and CA199 level before and after the operation did not impact the clinical outcome (*p* > 0.05). Multivariate analysis suggested that a D_90_ higher than 110 Gy and an age younger than 60 years were independent, favorable prognostic factors with a relative risk ratio of 0.21 and 0.34, respectively. Therefore, we recommend that the optimal dose for ^125^I seed implantation in patients with unresectable pancreatic cancer is at least 110 Gy.

## Conclusions

Intraoperative ultrasound-guided permanent ^125^I seed implantation is a safe, effective radiation technique for the treatment of unresectable pancreatic cancer. The technique provides satisfactory distribution of seeds within the tumor mass and achieves favorable clinical outcomes with acceptable complications. Additional studies with larger patient cohorts are now required in order to verify these results.

## Abbreviations

125I: Iodine-125; LDR: Low-dose rate; HDR: High-dose rate; IMRT: Intensity-modulated radiotherapy; EBRT: External beam radiotherapy; IORT: Intraoperative radiotherapy; GTV: Gross tumor volume; PTV: Planning tumor volume; SBRT: Stereotactic body radiation therapy; CR: Complete response; PR: Partial response; PD: Progressive disease; NRS: Numerical rating scale; KPS: Karnofsky performance status; RR: Relative risk.

## Competing interests

The authors declare that they have no competing interests.

## Authors’ contributions

JJW conceived, designed, coordinated the study and wrote the paper; HW, YLJ, JNL, SQT and YG contributed to the data collection and performed the statistical analysis; WQR and DRX performed the research. All authors read and approved the final version of the manuscript.

## Supplementary Material

Additional file 1: Table S1 Characteristics of Patients and Treatment. Click here for file

Additional file 2: Table S2 Results using intraoperative ultrasound‒guided implantation of 125I seeds for patients with locally advanced unresectable pancreatic cancer. Click here for file
