# Supplementary material for: The investigation of 125I seed implantation as a salvage modality for unresectable pancreatic carcinoma
Source: J Exp Clin Cancer Res. 2013 Dec 27;32(1):106. doi: 10.1186/1756-9966-32-106 (PMC3892087; doi:10.1186/1756-9966-32-106)
Supplement: Additional file 1: Table S1 — Characteristics of Patients and Treatment. [file 1756-9966-32-106-S1.pdf]

Additional Table 1.

Characteristics of Patients and Treatment

No	Gender	Age	Stage	TNM stage	Location	KPS	Jaundice	Lesion dimensions (cm)	Surgery	Seed activity (mCi)	Number of seeds	Adjuvant EBRT	Adjuvant CT (number of cycles)	Other treatment
1	M	53	II	T3N0M0	Head	80	Yes	3.0×3.0×3.0	No	0.4	23	No	No	PTCD+Stent
2	F	71	II	T3N0M0	Head	80	No	3.9×2.8×2.8	No	0.4	24	No	No	PTCD+Stent
3	F	43	II	T3N0M0	Whole	90	No	3.0×4.0×5.0	No	0.5	73	50Gy/25f	GEM(6)	No
4	F	66	II	T3N1M0	Head	80	No	3.0×4.0×4.0	No	0.5	28	No	No	No
5	F	38	II	T3N1M0	Head	70	Yes	4.0×4.0×4.0	Biliary enteric anastomosis	0.5	38	No	No	PTCD+Stent
6	F	64	II	T3N0M0	Head	80	Yes	4.5×3.0×3.0	Biliary enteric anastomosis +gastroiejunostomy	0.4	19	35Gy/17f	PTX(2)	No
7	M	65	II	T3N0M0	Head	80	Yes	5.0×5.0×4.5	Biliary enteric anastomosis	0.6	34	No	No	No
8	M	75	II	T3N0M0	Head	70	Yes	3.0×3.0×3.0	Biliary enteric anastomosis	0.5	10	No	No	No
9	M	54	II b	T2N1M0	Head	80	Yes	5.5×3.0×4.5	No	0.5	51	No	No	No
10	M	71	III	T4N0M0	Body	80	Yes	4.8×4.0×2.5	No	0.49	50	No	GEM(1)	PTCD+Stent
11	M	46	III	T4N0M0	Whole	70	No	5.0×8.0×5.0	No	0.5	75	40Gy/20f	GEM(4)	No
12	F	56	III	T4N0M0	Body	80	No	4.0×4.0×4.0	No	0.5	49	No	GEM(3)	No
13	M	52	III	T4N1M0	Body	80	No	6.0×5.0×4.0	No	0.6	44	50Gy/25f	No	No
14	F	48	III	T4N0M0	Head	80	Yes	4.1×4.0×4.0	Biliary enteric anastomosis +gastroiejunostomy	0.5	28	40Gy/20f	GEM(2) PTX(2)	No
15	M	62	III	T3N1M0	Head	70	Yes	2.5×3.0×3.3	Biliary enteric anastomosis	0.5	20	No	GEM(1)	No

**16**	**M**	**41**	Ⅲ	**T4N0M0**	**Head**	**80**	**No**	**5.0×4.0×4.0**	**No**	**0.5**	**58**	**No**	**No**	**No**
**17**	**F**	**56**	Ⅲ	**T4N1M0**	**Body**	**80**	**No**	**6.5×2.6×2.0**	**No**	**0.5**	**48**	**No**	**GEM(6)**	**No**
**18**	**M**	**46**	Ⅲ	**T4N0M0**	**Head**	**80**	**No**	**3.0×2.0×2.0**	**No**	**0.46**	**33**	**50Gy/25f**	**No**	**No**
**19**	**M**	**90**	Ⅲ	**T3N1M0**	**Body**	**70**	**No**	**6.5×4.5×2.5**	**No**	**0.5**	**45**	**No**	**No**	**No**
**20**	**M**	**43**	Ⅲ	**T4N0M0**	**Body**	**80**	**Yes**	**4.0×4.0×4.0**	**No**	**0.5**	**31**	**No**	**No**	**PTCD+Stent**
**21**	**M**	**62**	Ⅲ	**T4N1M0**	**Head**	**70**	**Yes**	**3.8×3.7×5.7**	**Biliary enteric anastomosis +gastroiejunostomy**	**0.5**	**48**	**45Gy/25f**	**No**	**No**
**22**	**M**	**63**	Ⅲ	**T4N0M0**	**Head**	**80**	**No**	**5.0×3.0×2.5**	**Biliary enteric anastomosis +gastroiejunostomy**	**0.4**	**55**	**No**	**No**	**No**
**23**	**F**	**59**	Ⅲ	**T4N1M0**	**Body**	**80**	**No**	**1.7×2.5×2.7**	**No**	**0.5**	**18**	**No**	**GEM(3)**	**No**
**24**	**F**	**54**	Ⅲ	**T4N1M0**	**Body**	**70**	**No**	**4.0×4.0×4.0**	**No**	**0.5**	**37**	**No**	**GEM(4)**	**No**
**25**	**M**	**73**	Ⅲ	**T4N1M0**	**Head**	**80**	**No**	**5.0×2.5×1.5**	**No**	**0.4**	**24**	**No**	**No**	**No**
**26**	**F**	**46**	Ⅲ	**T4N0M0**	**Body**	**80**	**No**	**3.7×3.2×2.7**	**No**	**0.5**	**56**	**No**	**No**	**No**
**27**	**M**	**78**	Ⅲ	**T4N0M0**	**Body**	**80**	**No**	**3.9×2.7×1.5**	**No**	**0.5**	**34**	**No**	**No**	**No**
**28**	**F**	**55**	Ⅲ	**T4N1M0**	**Body**	**80**	**No**	**2.0×2.0×2.5**	**No**	**0.4**	**12**	**No**	**No**	**No**

Abbreviations: KPS = Karnofsky Performance Status, EBRT = External Beam Radiotherapy, CT = Chemotherapy, PTCD = Percutaneous Transhepatic Choledochus Drainage, GEM = Gemcitabine, PTX = Paclitaxel.
