# Supplementary material for: The investigation of 125I seed implantation as a salvage modality for unresectable pancreatic carcinoma
Source: J Exp Clin Cancer Res. 2013 Dec 27;32(1):106. doi: 10.1186/1756-9966-32-106 (PMC3892087; doi:10.1186/1756-9966-32-106)
Supplement: Additional file 2: Table S2 — Results using intraoperative ultrasound‒guided implantation of 125I seeds for patients with locally advanced unresectable pancreatic cancer. [file 1756-9966-32-106-S2.pdf]

Additional Table 2.

Results using intraoperative ultrasound-guided implantation of ^{125}I seeds for patients with locally advanced unresectable pancreatic cancer

No	D_{90} (Gy)	NRS of pre-seed implant	NRS of post-seed implant	Response rate (pain)	Response rate (tumor)	Local control (months)	Overall survival (months)	Cause of death
1	120	No	No		CR	28	28	M
2	110	Moderate	No	Good Response	CR	24	24	M
3	140	Moderate	No	Good Response	PR	48	48	M
4	115	Severe	No	Good Response	PR	6	9	M+LR
5	120	No	No		PR	10	10	M
6	80	Severe	Mild	Medium Response	PD	3	8	LR
7	115	Severe	Mild	Medium Response	PR	5	5	Heart Disease
8	60	Moderate	No	Good Response	PR	3	3	M
9	140	No	No		PR	9	9	M
10	163	No	No		PR	7	7	N/A
11	120	No	No		CR	14	14	M
12	130	Moderate	Mild	Medium Response	PR	14	14	M
13	120	Severe	Moderate	Poor Respons	PR	13	13	M
14	80	Severe	Mild	Medium Response	PD	3	9	LR+M
15	125	No	No		PD	3	8	LR
16	140	Severe	No	Good Response	PR	11	11	M
17	151	No	No		PR	14	14	M
18	115	Severe	No	Good Response	SD	13	13	M
19	119	No	No		PR	10	10	M
20	106	Severe	Mild	Medium Response	PR	9	9	M
21	121	No	No		PR	9	9	M
22	161	Moderate	No	Good Response	PR	9	9	M
23	160	Severe	Mild	Medium Response	SD	10	10	M
24	94	No	No		PR	8	8	M
25	95	No	No		SD	5	5	M
26	97	Moderate	No	Good Response	PR	10	10	M

**27**	**162**	**Moderate**	**Mild**	**Medium Response**	**PR**	**11**	**11**	**M**
**28**	**93**	**Severe**	**Mild**	**Medium Response**	**PR**	**5**	**5**	**M**

Abbreviations: D_{90} = The reference dose that at least 90% of the tumor volume received, NRS = Numerical rating scale, CR = Complete Response, PR = Partial Response, SD = Stable Disease, PD = Progressive Disease, LR = Local Recurrence, M = Metastasis.
